# Curcumin Restrains Oxidative Stress of After Intracerebral Hemorrhage in Rat by Activating the Nrf2/HO-1 Pathway

**DOI:** 10.3389/fphar.2022.889226

**Published:** 2022-04-27

**Authors:** Chenyang Duan, Hanbin Wang, Dian Jiao, Yanqin Geng, Qiaoli Wu, Hua Yan, Chunhui Li

**Affiliations:** ^1^ Affiliated Hospital of Hebei University, Baoding, China; ^2^ Hebei University, Baoding, China; ^3^ Tianjin University, Tianjin, China; ^4^ Tianjin Huanhu Hospital, Tianjin University, Tianjin, China; ^5^ Tianjin Key Laboratory of Cerebral Vascular and Neurodegenerative Diseases, Tianjin Neurosurgical Institute, Tianjin Huanhu Hospital, Tianjin, China; ^6^ School of Medicine, Nankai University, Tianjin, China

**Keywords:** curcumin, intracerebral hemorrhage, microglia, hematoma, Nrf2, HO-1, ROS, oxidative stress

## Abstract

Intracerebral hemorrhage (ICH), a severe hemorrhagic stroke, induces cerebral oxidative stress and severe secondary neurological injury. Curcumin was demonstrated to inhibit oxidative stress in the brain after ICH. However, the pharmacological mechanism needs further research. We used an intrastriatal injection of autologous blood to make the rat ICH model, and then the rat was treated with curcumin (100 mg/kg/day). Modified Neurological Severity Score (mNSS) and corner test results showed that curcumin could significantly promote the neurological recovery of ICH rats. Meanwhile, curcumin could substantially reduce ROS and MDA in the tissues around intracranial hematoma and prevent GSH depletion. To explore the pharmacological molecular mechanism of curcumin, we used HAPI cells and primary rat cortical microglia for *in vitro* experiments. *In vitro*, heme-treated cells were used as the cell model of ICH to explore the molecular mechanism of inhibiting oxidative stress by curcumin treatment. The results showed that curcumin significantly inhibited heme-induced oxidative stress, decreased intracellular ROS and MDA, and promoted Nrf2 and its downstream antioxidant gene (HO-1, NQO1, and Gpx4) expression. These results suggest that curcumin inhibits oxidative stress by activating the Nrf2/HO-1 pathway. Here, our results indicate that curcumin can promote the inhibition of oxidative stress in microglia by activating the Nrf2/HO-1 pathway and promoting neurological recovery after ICH, providing a new therapeutic target for clinical treatment of ICH.

## Introduction

Intracerebral hemorrhage (ICH) is defined as bleeding in the brain parenchyma (Aguilar and Freeman, 2010) and has high disability and mortality rates (van Asch et al., 2010). In the past 40 years, the mortality rate of intracerebral hemorrhage has decreased (Sipilä et al., 2021), but the disability rate has not (Feigin et al., 2020), which may be related to nerve injury, secondary to intracerebral hemorrhage, and neuronal loss caused by oxidative stress is the main factor (Duan et al., 2016). After ICH, a hematoma can result in a rapid and continuous increase in intracranial reactive oxygen species (ROS), causing sustained intracranial oxidative stress and neuronal death, leading to secondary neurological impairment (Hu et al., 2016). Microglia are critical immune cells in the central nervous system. Several studies have shown that microglia can scavenge intracranial ROS and inhibit oxidative stress (Zhao et al., 2007b; Zhang et al., 2016; Jin et al., 2021). In recent years, microglia have been used to inhibit oxidative stress after ICH to rescue neurons and restore neural function as a new therapeutic strategy for ICH.

Nuclear factor erythroid 2-related factor 2 (Nrf2), a transcription factor activated by oxidative stress, increases the expression of antioxidants (Chen, 2021), including heme oxygenase-1 (HO-1), NAD(P)H: quinone oxidoreductase 1 (NQO1), catalase (CAT), glutathione S-transferase (GST), superoxide dismutase (SOD), and other genes. Although transcription factors other than Nrf2 can regulate these genes, Nrf2 plays an essential role in antioxidation and maintaining oxidative stress balance *in vivo* by regulating the expression of most of these genes (Giudice and Montella, 2006). Under physiological conditions *in vivo*, Nrf2 is sequestered in cells by Kelch-like ECH-associated protein 1 (Keap1), subsequently ubiquitinated and degraded. However, when ROS are increased, the affinity of Keap1 and Nrf2 is decreased, and Nrf2 enters the nucleus and binds with antioxidant responsive elements (AREs) to promote the transcription of downstream genes (Moi et al., 1994). Several studies have demonstrated that activation of the Nrf2 pathway can promote the recovery of neurological function after stroke and curb the progression of degenerative neurological diseases (Yao et al., 2016; Osama et al., 2020).

**FIGURE 1:**
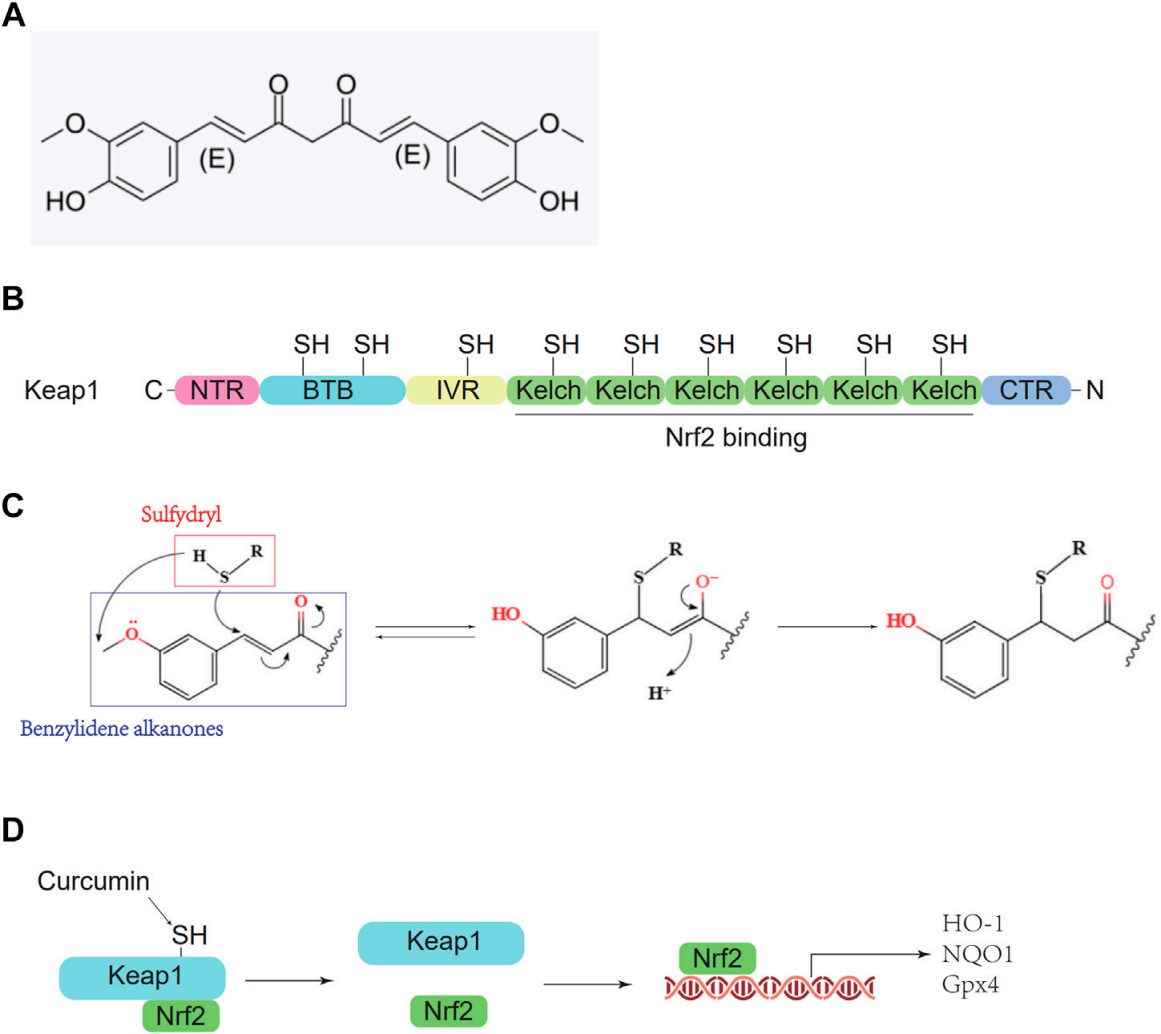
Pharmacological molecular mechanisms of curcumin. **(A)** The chemical structure of Curcumin. **(B)** Domain architecture of Keap1. **(C)** Chemical reaction sketch described curcumin’s enhanced reactivity of the SH group. **(D)** Curcumin inhibits Keap1 capture and binds Nrf2.

Curcumin, a polyphenolic substance, is one of the main active substances in the rhizomes of the perennial herb turmeric (Banerjee and Chakravarty, 2015) and is defined as 1, 7-bis(4-hydroxy3-methoxyphenyl)-1,6-heptadiene-3,5-dione (Figure 1A). Curcumin has antioxidative, anti-inflammatory, and anticancer effects. Although many experiments have shown that curcumin has significant effects on myocardial ischemia (Chen, 2021), hepatitis (Leclercq et al., 2004), and ischemic stroke (Yao et al., 2016), the molecular mechanism still needs further study. Curcumin contains benzylidene alkanone groups with high thiol affinity, and the amino acid residues in the Keap1 protein have nine thiol groups (Figure 1B). Therefore, curcumin can react with the sulfhydryl group in the Keap1 protein to break the hydrogen bond in Keap1 (Figure 1C), thereby reducing the affinity of Keap1 and Nrf2 so that Nrf2 can enter the nucleus and bind with AREs to promote downstream gene transcription (Figure 1D) (Dinkova-Kostova et al., 2001). To determine whether curcumin plays an antioxidant role by promoting the Nrf2 pathway, ML385 is used as a specific Nrf2 inhibitor in this study. ML385 can disrupt the dimerization of Nrf2 and MAFG (a small molecule Maf family protein) and decrease the stability of Nrf2 binding to AREs, inhibiting downstream gene transcription (Singh et al., 2016). This study demonstrates the molecular mechanism and related cellular pathways of curcumin-mediated inhibition of oxidative stress in ICH, which is conducive to further development and research of curcumin-based drugs and promotes the use of curcumin in the clinical treatment of ICH.

## Materials and Methods

### Experimental Animals

Male Sprague–Dawley (SD) rats (China National Institute for Food and Drug Control) weighing–250–300 g and aged approximately 10 weeks were kept in a constant temperature experimental cage with a temperature of 20–25°C and humidity of 50–60%. There were up to four rats in each cage. The cage bedding was changed twice a week to ensure that the rats’ living environment was dry and clean. The rats were fed special nutritional feed to ensure adequate feed and water. Appropriate (12 h of light and 12 h of darkness) circadian light cycles were provided. All animal experiments are approved by the Animal Experiment Ethics Committee of Tianjin Huanhu Hospital (TJ.546620201207) and implemented in accordance with NIFDC guidelines.

### Rat ICH Model

An intracranial injection of autologous blood was used to simulate human ICH in the rats, as we previously described (Wang et al., 2018). In brief, as preoperative preparation, Sprague–Dawley rats (SPF grade; 240–280 g) were fasted overnight before ICH was established. Initially, each rat was anesthetized with 2% pentobarbital (0.2 ml/100 g). The rats underwent surgery using an ultraclean table and were fixed in a stereotaxic frame. The scalp was opened to expose the anterior brain region. A dental drill was used to drill a 1-mm-diameter hole in the skull surface. Blood (100 μl) was collected from the rat tail vein and injected into the rat striatum with a microsyringe (stereotaxic coordinates; 2 mm lateral to the midline, 0.2 mm posterior to bregma, and 5.5 mm deep below the skull). First, 60 μl of autogenous blood were injected at a rate of 2 μl/min, and the next 40 μl of blood were injected at 5 μl/min. Finally, the needle was left for 10 min before being removed. In postoperative care, the coronal aponeurosis and scalp tissue were sutured. The rats were returned to their cage and provided adequate water and feed. If the rats’ mobility was greatly impaired, the feed was placed in the cage to ensure that the animals could access the feed.

### Neurological Function Test

An independent researcher who was blinded to the experimental design performed the neurological function test on days 1, 2, 3, 5, and 7 after ICH.

### Modified Neurological Severity Score

The mNSS was performed as previously described (Chen et al., 2001). According to the mNSS, neurological functions, including motor and sensory systems, reflexes, and balance, were graded on a numeric scale from 0 to 18 (the maximal mNSS was 18, indicating maximum neurological impairment, and the minimal mNSS was 0, indicating the absence of impairment).

### Brain Water Content Measurements

Cerebral edema in each group of rats was assessed using the wet-dry method as previously described (Zeng et al., 2020). In brief, 24 h after ICH induction, the rats were anesthetized with 2% pentobarbital (0.2 ml/100 g) intraperitoneally and euthanized by cervical dislocation. The two hemispheres of the brain were separated from the midline and then divided into two parts. After being weighed (considered the wet weight) and recorded separately, the brain tissues were dried for 48 h at 100°C and weighed again (considered the dry weight). The following formula was used to assess the brain water content: water content = [(wet weight − dry weight)/wet weight] 100%.

### Cell Culture

The microglial cell line HAPI (American Type Culture Collection (ATCC), Manassas, VA, United States) was cultured in DMEM with 10% FBS, 100 U/ml penicillin, and 100 mg/ml streptomycin and then placed in a 37°C humidified incubator with 5% CO_2_.

### Primary Cortical Microglial Cultures

The primary cortical microglial culture of newborn rats has been described in detail in the previous literature (Lin et al., 2017). In brief, the newborn rats were sacrificed and immersed. They were sterilized in 75% alcohol, then the cortical tissue of the fetal brain of the mouse was taken out under a dissecting microscope, and was put into the DMEM medium (11995040, Gibco, Shanghai, China) containing 1% Penicillin-Streptomycin Solution (15140122, Gibco, Shanghai, China) and Cell Culture Sera (all operations are performed on an ice box). It is digested with 0.05% Pancreatin at 37°C for 10 min, filtered through a 75-micron sieve, and then centrifuged three times (800, 1,000,, and 1,200 rpm) for 5 min each to remove cell debris and impurities. According to the cell density of 3 × 10^5^/well, the plate is generally plated in a 24-well plate that has been precoated with polylysine. After 24 h, the mixed DMEM medium was used to culture until 4 days. And then, the culture medium supernatant was removed and cultured in a T25 cell culture flask, and the experiment was carried out after two generations.

### Detection of MDA and Reduced Glutathione

The markers MDA and glutathione, which can indicate the degree of oxidative stress injury, were extracted and detected by the MDA kit (BC0025, Solarbio, Beijing, China) and glutathione kit (BC1175, Solarbio, Beijing, China). The MDA and reduced glutathione contents of the processed brain tissue, HAPI cells, and primary microglia passed the detection. The kit was tested according to the manufacturer’s instructions.

### Intracellular ROS Measurements

HAPI cells and microglia were planted in a 12-well plate and then treated with varying concentrations of the test compound or drug. After 12 h, cells were incubated at a final concentration of 5 µM DCFH-DA (WLA131, wanleibio, Dalian, China) for 30 min at 37°C, after which they were washed, dyed with DAPI, and immediately analyzed for fluorescence intensity under a laser confocal microscope with a ×20 objective lens.

### Flow Cytometry

12 h after adding the CFSE-labeled RBCs to the HAPI cells, cells were incubated at a final concentration of 5 µM DCFH-DA (WLA131, wanleibio, Dalian, China) for 30 min at 37°C, after which they were washed. Then, a PBS suspension of the HAPI cells was obtained for the flow cytometry analysis. The intracellular ROS was analyzed using a flow cytometer. The fluorescence intensity of FITC in flow cytometry results evaluated intracellular ROS levels.

### Western blot

A WB analysis was performed as previously described (Yang et al., 2021). Cerebral tissues and cells were lysed with RIPA lysis buffer. The protein concentration was determined using an enhanced BCA protein assay kit. The total protein (20 μg) from each sample was separated by SDS–PAGE and transferred onto a polyvinylidene difluoride (PVDF) membrane. The nonspecific binding sites on the PVDF membrane were blocked by incubation with 5% nonfat milk in Tris-buffered saline-Tween (TBST) for 1 h. Then, the PVDF membrane was incubated overnight at 4°C with antibodies against Nrf2 (1:1,000, #12721, Cell Signaling Technology), HO-1, NQO1, Gpx4, SOD, Caspase 8, and Tubulin (1: 1,000, #2128, Cell Signaling Technology). Then, the membrane was washed with TBST and incubated with horseradish peroxidase-conjugated horse antimouse IgG for 1 h at room temperature. Immunoreactivity was visualized using an enhanced chemiluminescence kit. The band intensities were quantified by a densitometric analysis using Image J software.

### RT-qPCR

A RT-qPCR analysis was performed as previously described (Wilkerson et al., 2013). The brains were removed and then perfused with PBS. The 2 mm ipsilateral striatum surrounding the hematoma was removed on ice and washed with cold PBS. Then, the total RNA was extracted using TRIzol, and the extracted RNA was reverse transcribed into cDNA by a reverse transcription kit. The qPCR was performed on a Roche Lightcycler II using the target gene and the reference GAP gene primers. The Fold change result was calculated by the 2^−ΔΔCT^ (Livak) method. The primer sequences designed to detect specific genes are listed in the Supplementary Material.

### Immunocytochemistry

We performed immunocytochemical staining according to the methods described in a previous study (Zhao et al., 2020). The rat brain tissue was perfused with PBS and PFA. After anesthesia with pentophenobarbital, the thoracic cavity of the rat was cut open, and a disposable blood needle was inserted into the left ventricle at the apex of the heart, while the right auricle was cut open. After PBS perfusion, 4% PFA was used to continue perfusion and fix the tissue. Then, the brain of the rat was removed, fixed by soaking in PFA for 24 h, dehydrated by a gradient of 10, 20 and 30% sucrose solutions, and preliminarily cut into 0.5-cm-thick brain slices. After embedding in OCT, the tissue was placed in liquid nitrogen for rapid cooling. Then, frozen slices of brain tissue with a thickness of 10 μM were obtained by frozen sectioning at −20°C. After the frozen brain tissue sections were removed, the excess OCT glue was removed, and the frozen tissues were circled with crayons. Then, the frozen tissues were fixed with 4% PFA, and Tween-20 was added to increase the permeability of the cell membranes. Finally, after sealing with sheep serum, the primary antibodies were diluted in antibody diluents in different proportions [Nrf2 (1:100, #12721, Cell Signaling Technology) and HO-1], and the tissues were incubated at 4°C overnight. Then, different diluted fluorescent secondary antibodies were added, and the samples were incubated at 37°C for 40 min. Finally, a sealing tablet containing DAPI was added to seal the slides. The fluorescence images were obtained under a confocal microscope.

### Statistical Analysis

Data are expressed as mean ± SD. Statistical analysis was performed using the ANOVA test followed by the Newman–Keuls multiple-comparison post hoc test. *p* < 0.05 was considered statistically significant.

## Results

### Nrf2/HO-1 Is the Apical Pathway Activated Upon Oxidative Stress in HAPI Cells

**FIGURE 2:**
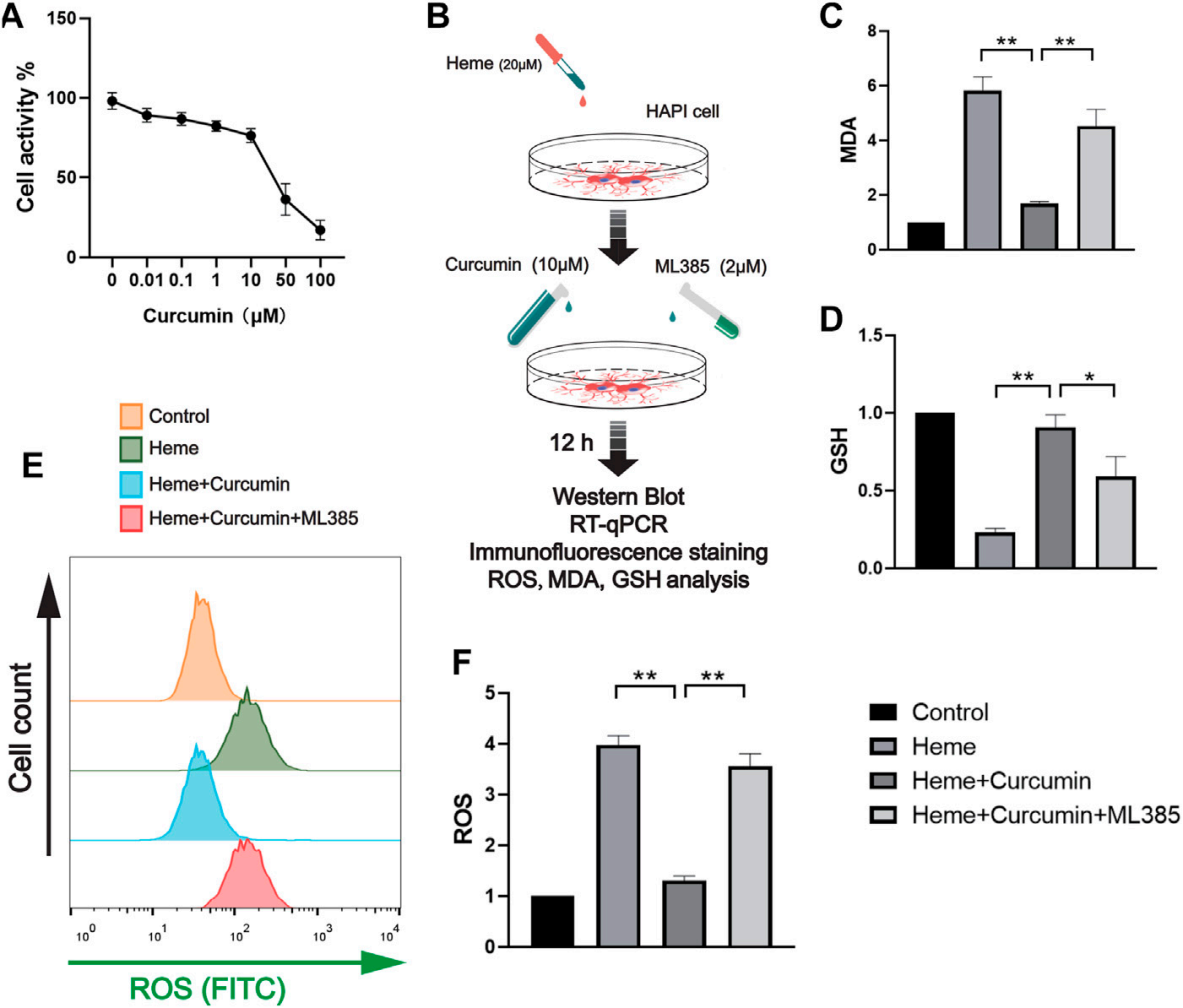
Curcumin was confirmed to promote the antioxidant function of HAPI cells. **(A)** The activity of the HAPI cells treated with curcumin at different concentrations for 12 h was determined, and the cellular activity of the cells was evaluated by the CCK-8 analysis. **(B)** *In vitro* experimental procedures. **(C,D)** Based on the CCK-8 analysis, the heme-induced HAPI cells were treated with curcumin (10 μM) for 12 h, then the levels of MDA and GSH were determined. **(E)** After ROS in each group were labeled by FITC, flow cytometry results were performed. **(F)** ROS levels in HAPI cells were measured with a microplate reader. Data are shown as the means ± SD of *n* = 3 samples/group and at least three independent experiments were performed. Statistical analyses were conducted using two-way ANOVA. *, *p* < 0.01 and **, *p* < 0.01.

We treated the HAPI cells with different concentrations of curcumin for 12 h and then analyzed cell activity using CCK-8 (Figure 2A). The required concentration of curcumin was determined to be 10 μM. The cells were divided into four groups as follows: control group, heme group (treated with heme 50 μM, then cultured for 12 h), heme + curcumin group (treated with heme 50 and 10 μM curcumin was added then cultured for 12 h), and heme + curcumin + ML385 group (treated with Heme 20 μM, curcumin 10 μM, and ML385 2 μM were added simultaneously). Then cultured for 12 h. In the HAPI cells, curcumin could inhibit the increase of MDA level in response to heme, while inhibition of the Nrf2 pathway by ML385 substantially reduced the inhibitory effect of curcumin on MDA level (Figure 2C). GSH, an important endogenous antioxidant, was remarkably depleted in response to oxidative stress caused by heme. However, curcumin could inhibit the depletion of GSH, meanwhile, inhibiting the Nrf2 pathway with ML385 only moderately affecting the effect of curcumin (Figure 2D). The curcumin-treated HAPI cells could effectively scavenge ROS produced by oxidative stress, while inhibition of the Nrf2 pathway made curcumin fail to prevent the increase of ROS (Figure 2E). Changes in the FITC fluorescence intensity of flow cytometry, which determine the ROS level in cells, also supported this conclusion (Figure 2F).

**FIGURE 3:**
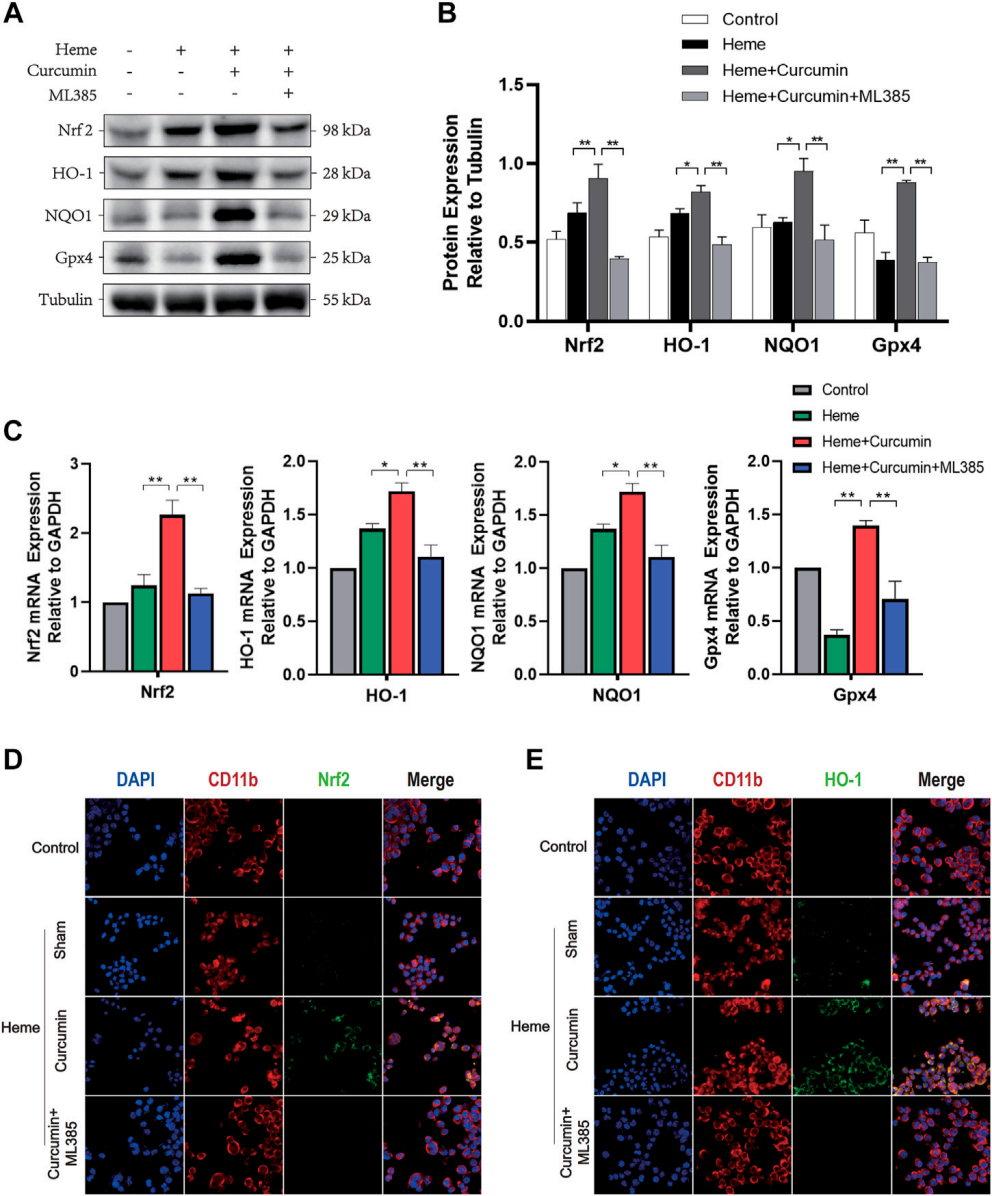
Curcumin could activate the Nrf2/HO-1 pathway in HAPI cells. **(A)** Western blot analysis of each group of HAPI cells. **(B)** ImageJ software was used to further quantify the gray values of Western blot bands. **(C)** The expression of the Nrf2, HO-1, NQO1, and Gpx4 was detected using RT–qPCR and compared with that of an internal reference gene (GAP). **(D)** The HAPI cell membrane protein CD11b was labeled with Texas Red, and Nrf2 was marked by FITC. **(E)** The HAPI cell membrane protein CD11b was labeled with Texas Red, and HO-1 was marked by FITC. The data are shown as the means ± SD of *n* = 3 samples/group and at least three independent experiments were performed. Statistical analyses were conducted using two-way ANOVA. *, *p* < 0.01 and **, *p* < 0.01.

We, therefore, studied the molecular mechanism of scavenging ROS and preventing oxidative stress during Nrf2 activation by curcumin. In the heme-treated HAPI cells, Western blot analyses identified a significant increase of Nrf2 in response to curcumin (Figure 3A). The amounts of HO-1, NQO1 and Gpx4, and several common antioxidant proteins, substantially increased likewise suggesting that Nrf2 activation promoted the expression of multiple antioxidant proteins (Figures 3B,C). Changes in the fluorescence intensity of Nrf2 and HO-1 proteins supported this conclusion (Figures 3D,E). Overall, this indicated that curcumin-induced Nrf2 activation could effectively promote the expression of downstream antioxidant genes, therefore, significantly inhibiting the oxidative injury of the HAPI cells induced by heme.

### Nrf2/HO-1 Is the Apical Pathway Activated Upon Oxidative Stress in Rat Primary Microglia

**FIGURE 4:**
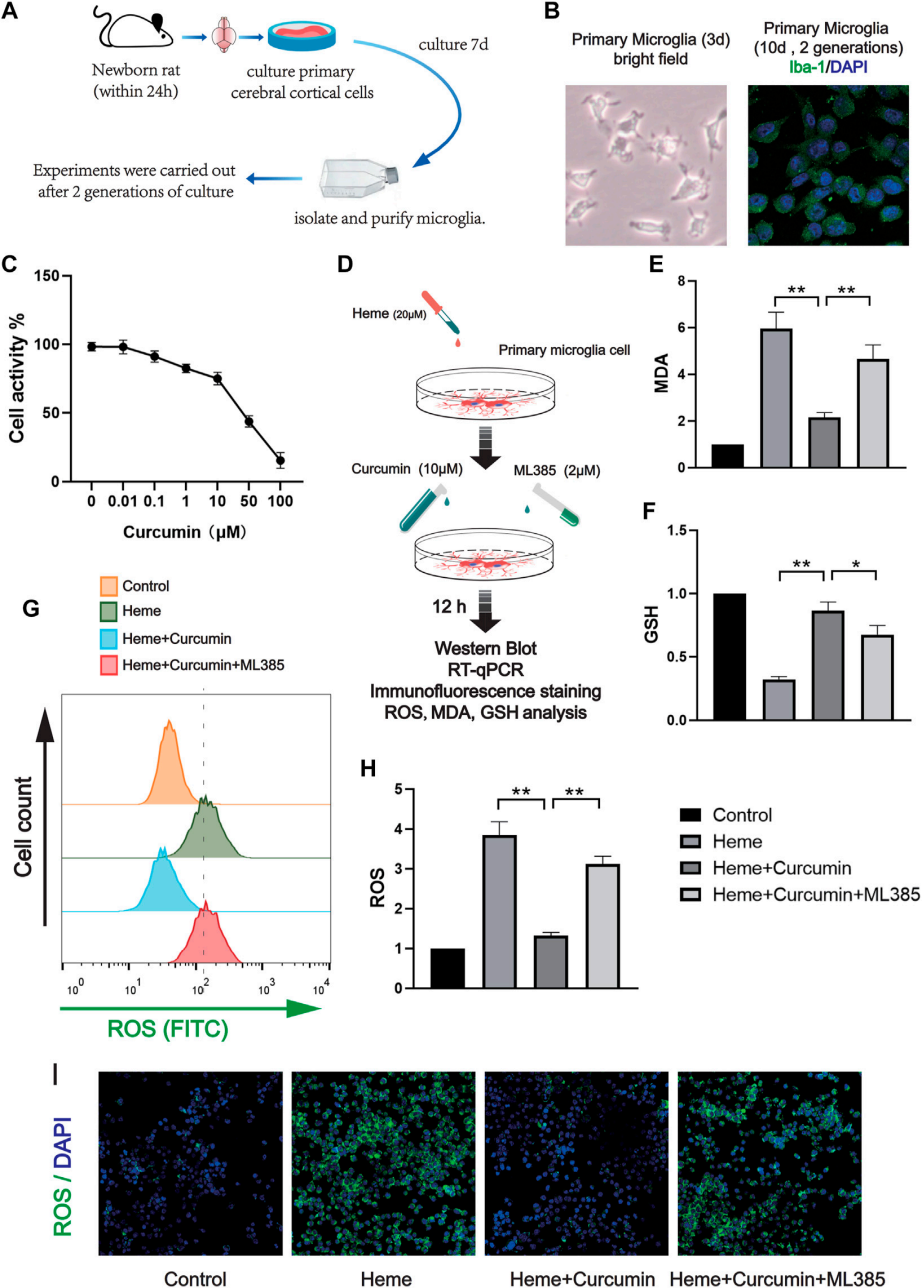
Curcumin was confirmed to promote the antioxidant function of rat primary microglia. **(A)** The activity of the primary microglia treated with curcumin at different concentrations for 12 h was determined, and the cellular activity of the cells was evaluated by the CCK-8 analysis. **(B)** *In vitro* experimental procedures. **(C,D)** Based on the CCK-8 analysis, heme-induced primary microglia were treated with curcumin (10 μM) for 12 h, then the levels of MDA and GSH were determined. **(E)** After ROS in each group were labeled by FITC, flow cytometry results were performed. **(F)** ROS levels in primary microglia were measured with a microplate reader. **(I)** Intracellular ROS levels were analyzed using the ROS Assay Kit and the results were presented using confocal fluorescence images, in which FITC labeled intracellular ROS. The data are shown as the means ± SD of *n* = 3 samples/group and at least three independent experiments were performed. The statistical analyses were conducted using the two-way ANOVA. *, *p* < 0.01 and **, *p* < 0.01.

In order to make the experimental results accurate and credible, we cultured rat primary microglia for the *in vitro* experiments. After two times of passage, the primary microglial cells were highly purified for the experiments. Similar to the previous experiment with the HAPI cells, we treated the primary microglial cells with different concentrations of curcumin for 12 h and then analyzed cell activity using CCK-8. The required concentration of curcumin was determined to be 10 μM (Figure 4A). The cells were divided into four groups as follows: control group, heme group (treated with heme 50 μM, then cultured for 12 h), heme + curcumin group (treated with heme 50–10 μM curcumin was added then cultured for 12 h), and heme + curcumin + ML385 group (treated with heme 20 μM, curcumin 10 μM, and ML385 2 μM were added simultaneously. Then cultured for 12 h) (Figure 4B). In the primary microglia, curcumin could inhibit the increase of MDA level in response to heme, while inhibition of the Nrf2 pathway by ML385 substantially reduced the inhibitory effect of curcumin on MDA level (Figure 4C). GSH, an important endogenous antioxidant, was remarkably depleted in response to oxidative stress caused by heme. However, curcumin could inhibit the depletion of GSH, meanwhile, inhibiting the Nrf2 pathway with ML385 only moderately affecting the effect of curcumin (Figure 4D). Curcumin-treated primary microglial cells could effectively scavenge ROS produced by oxidative stress, while inhibition of the Nrf2 pathway made by curcumin failed to prevent the increase of ROS (Figure 4E). Changes in the FITC fluorescence intensity of flow cytometry, which determine ROS level in cells, also supported this conclusion (Figure 4F). Moreover, confocal microscopy was used to photograph the primary microglia treated with the ROS analysis kit to observe intracellular ROS labeled by FITC. The result shows that in the primary microglia, curcumin could scavenge ROS produced by heme (Figure 4I). The curcumin-treated primary microglial cells could effectively scavenge ROS produced by oxidative stress, while inhibition of the Nrf2 pathway made Curcumin fail to prevent the increase of ROS (Figure 4G). Changes in the FITC fluorescence intensity of flow cytometry, which determine the ROS level in cells, also supported this conclusion (Figure 4H).

**FIGURE 5:**
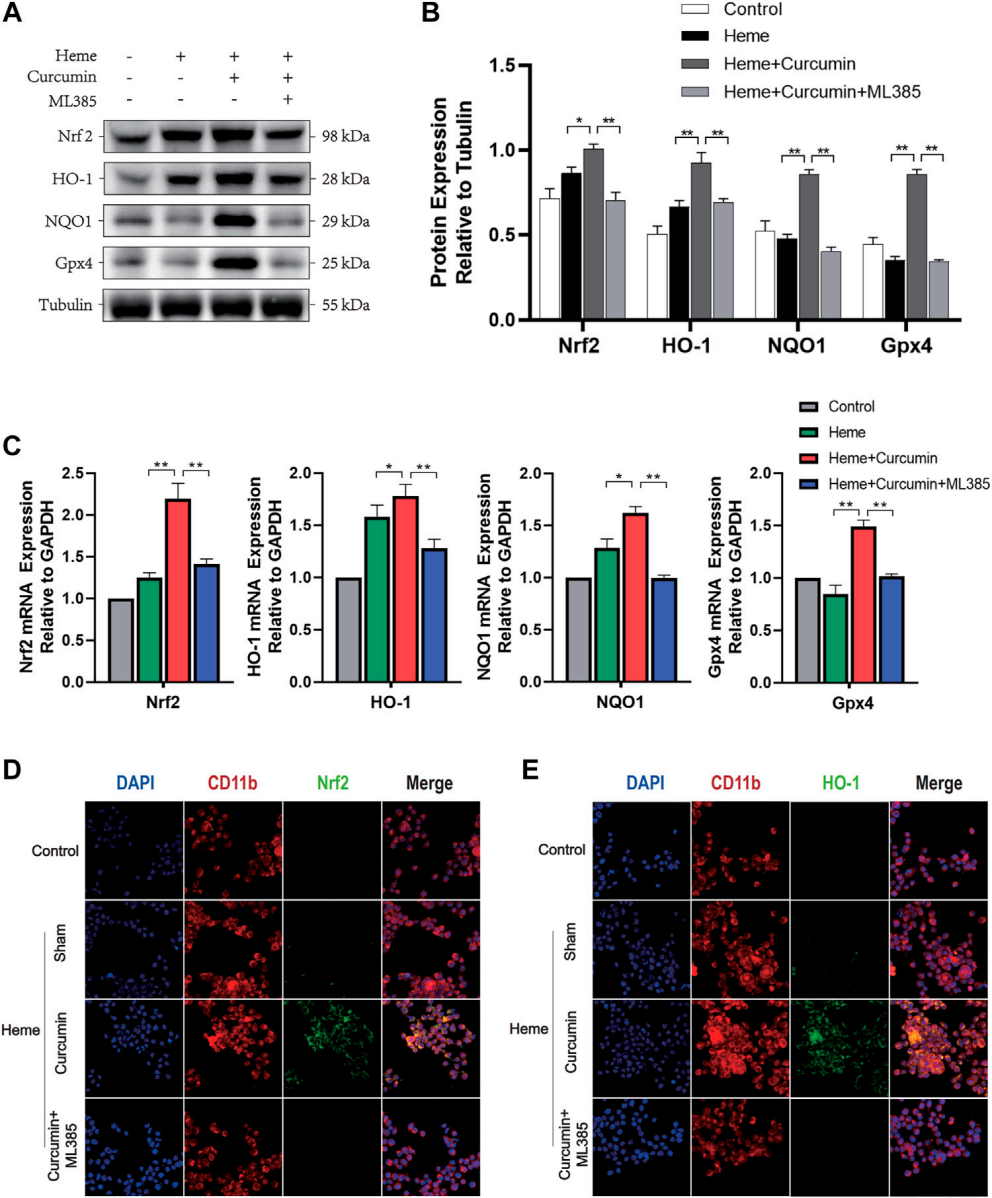
Curcumin could activate the Nrf2/HO-1 pathway in rat primary microglia. **(A)** Western blot analysis of each group of primary microglia. **(B)** ImageJ software was used to further quantify the gray values of the western blot bands. **(C)** The expression of the Nrf2, HO-1, NQO1, and Gpx4 was detected using RT–qPCR and compared with that of an internal reference gene (GAP). **(D)** The primary microglial membrane protein CD11b was labeled with Texas Red, and Nrf2 was marked by FITC. **(E)** The primary microglial membrane protein CD11b was labeled with Texas Red, and HO-1 was marked by FITC. The data are shown as the means ± SD of *n* = 3 samples/group and at least three independent experiments were performed. The statistical analyses were conducted using the two-way ANOVA. *, *p* < 0.01 and **, *p* < 0.01.

We, therefore, studied the molecular mechanism of scavenging ROS and preventing oxidative stress during Nrf2 activation by curcumin. In the heme-treated primary microglial cells, the Western blot analysis identified a significant increase of Nrf2 in response to curcumin (Figure 5A). The amounts of HO-1, NQO1, Gpx4, and several common antioxidant proteins, substantially increased, likewise suggesting that Nrf2 activation promoted the expression of multiple antioxidant genes. (Figures 5B,C) Changes in the fluorescence intensity of Nrf2 and HO-1 proteins supported this conclusion (Figures 5D,E). Overall, this indicated that curcumin-induced Nrf2 activation could effectively promote the expression of downstream antioxidant genes, therefore, significantly inhibiting the oxidative injured of primary microglia induced by heme.

### Curcumin Confers the Recovery of Neurological Function Recovery After ICH

**FIGURE 6:**
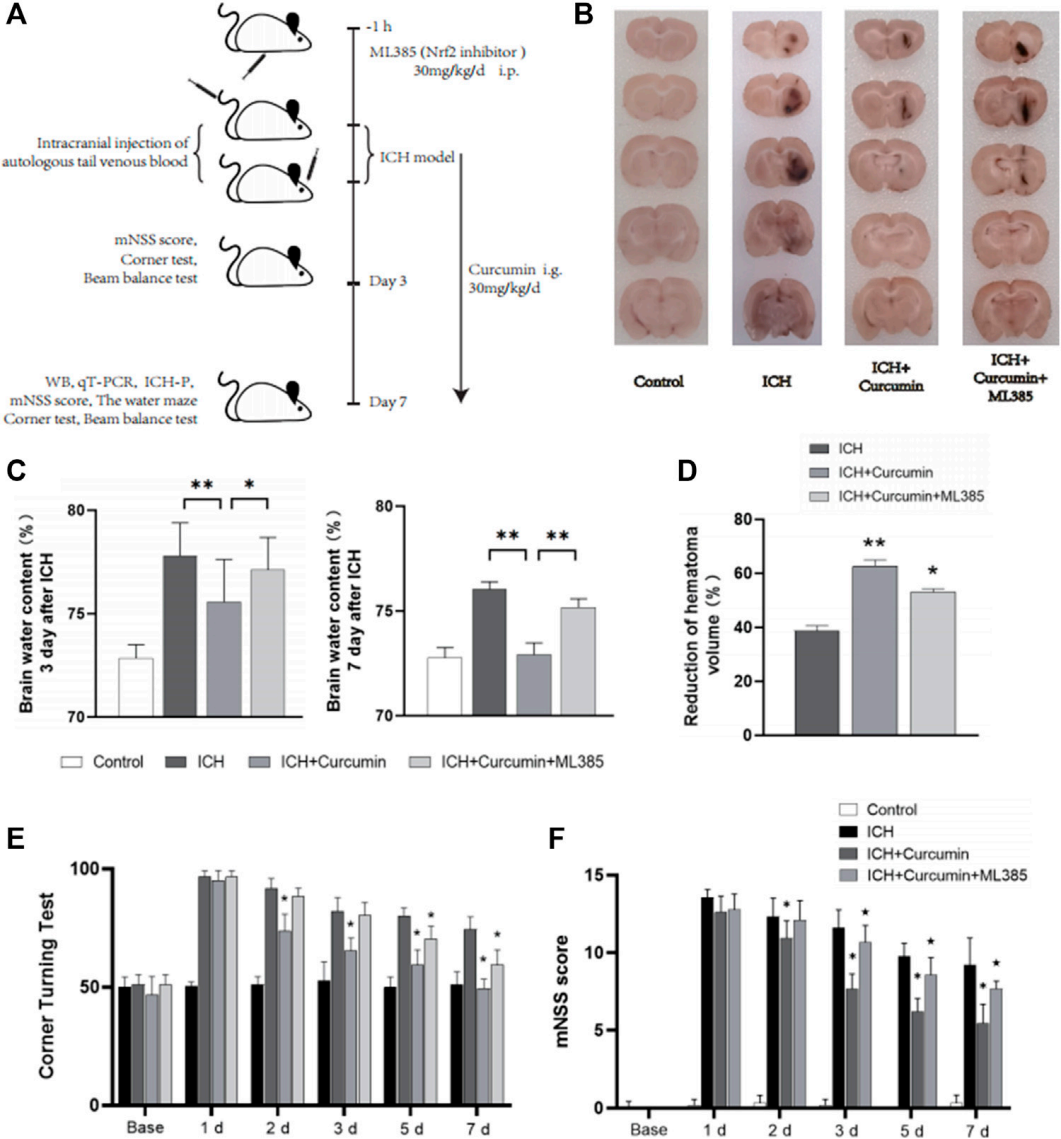
*In vivo* experimental procedures and neurological function evaluation. **(A)** Graphical abstract of the *in vivo* study. **(B)** The coronal sections of the brain tissue were prepared following sucrose dehydration (5 mm), to evaluate intracranial hematoma. **(C)** The cerebral water content was measured 3 and 7 d after ICH. **(D)** The reduction in the hematoma volume is reported as a percentage. **(E)** Statistical analysis of the corner turn test. **(F)** The mNSS score of rats in each group. The data are shown as the means ± SD of *n* = 3 samples/group and at least three independent experiments were performed. Statistical analyses were conducted using two-way ANOVA. Compared with ICH group, *, *p* < 0.01 and **, *p* < 0.01. Compared with ICH + Curcumin group, ★, *p* < 0.01.

In order to demonstrate intracellular ROS-scavenging effect of curcumin, we used an intrastriatal injection of autologous blood to make a rat ICH model for the *in vivo* experiments. 24 rats were randomly divided into four groups, and the rats in each group were treated differently. Control group and ICH group: 100 μl of autogenous blood was injected into the striatum of rats. ICH + Curcumin group: curcumin 100 mg/kg/day intragastric administration after ICH, and the first dose was administered 2 h after ICH. ICH + Curcumin + ML385 group: ML385 (30 mg/kg/day, i.p.) was administered 1 h before the administration of curcumin (Figure 6A). The mNSS neurological function score and Angle test were performed at 1d, 2d, 3d, 5d, and 7 days before and after ICH to evaluate the neurological function recovery of the rats. The results showed that curcumin could significantly improve the behavioral test results of ICH rats. (Figures 6E,F) At 7 days after ICH, the rat brain was sectioned into coronal sections 0.5 mm thick. The results showed that curcumin effectively promoted the clearance of intracranial hematoma, while ML385, an inhibitor of Nrf2, significantly affected the therapeutic effect of curcumin (Figures 6B–D). To evaluate the level of cerebral edema, the water content in the brain was analyzed. The results showed that curcumin decreased the water content of perihematomal brain tissue, and inhibition of Nrf2 by ML385 could significantly affect the therapeutic effect of curcumin (Figure 6C).

### The Activation Effect of Curcumin on Nrf2/HO-1 Is an Important Molecular Pharmacological Mechanism of Its Antioxidant Effect

**FIGURE 7:**
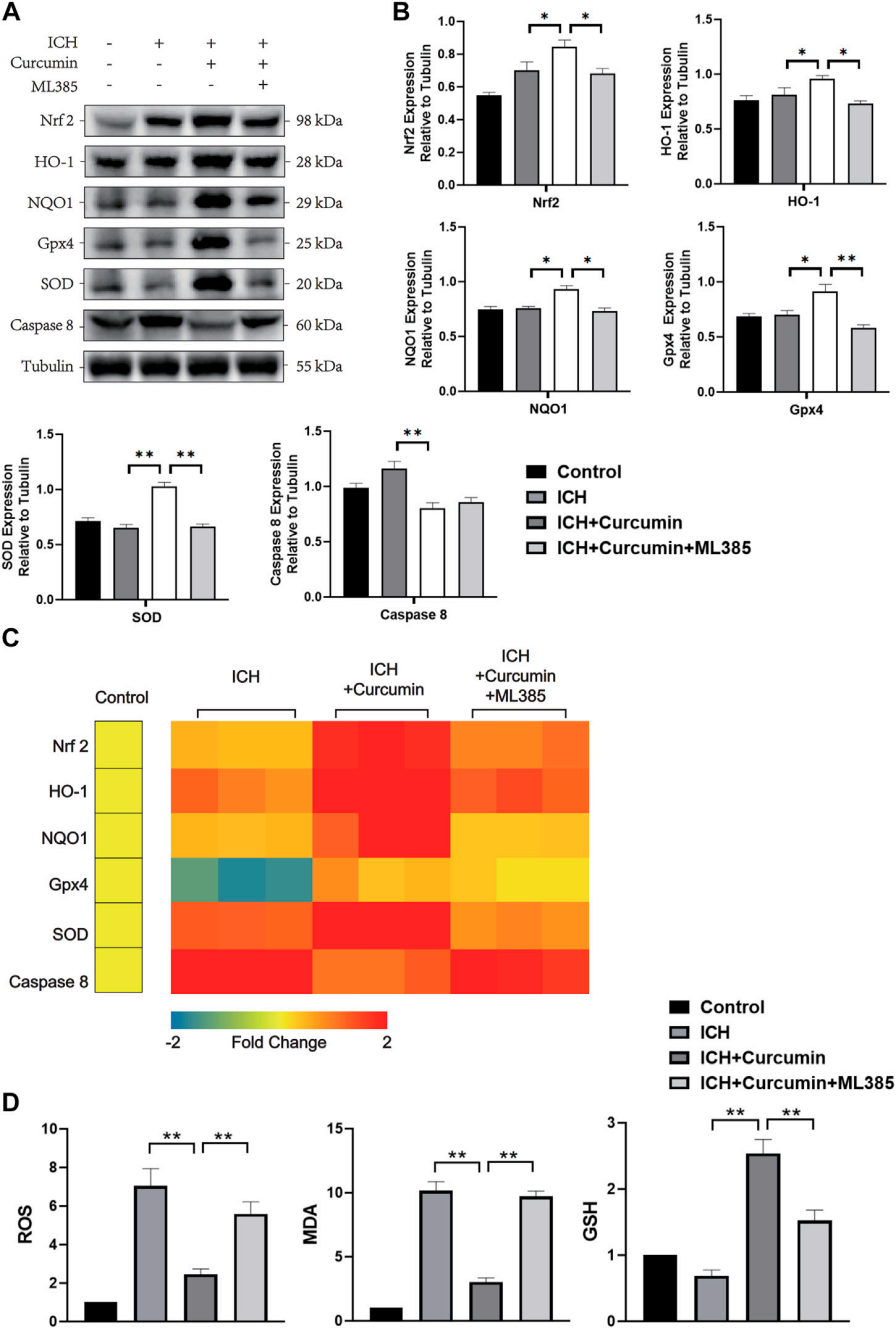
*In vivo* experiments were performed to evaluate the function of curcumin activation of the Nrf2/HO-1 pathway. **(A)** The western blot analysis of Nrf2, HO-1, NQO1, Gpx4, SOD, and Caspase8 in the surrounding tissues of hematoma at 7 d after ICH. **(B)** ImageJ software was used to further quantify the gray values of Western blot bands. **(C)** Heat map of the mRNA abundance of genes in the Nrf2 signaling pathway, including Nrf2, HO-1, NQO1, Gpx4, SOD, and Caspase8. **(D)** The kit was used to detect the MDA, ROS, and GSH content in each group of the surrounding tissues of hematoma (the specific detection method has been described in detail above). Data are shown as the means ± SD of *n* = 3 samples/group and at least three independent experiments were performed. The statistical analyses were conducted using the two-way ANOVA. *, *p* < 0.01 and **, *p* < 0.01.

**FIGURE 8:**
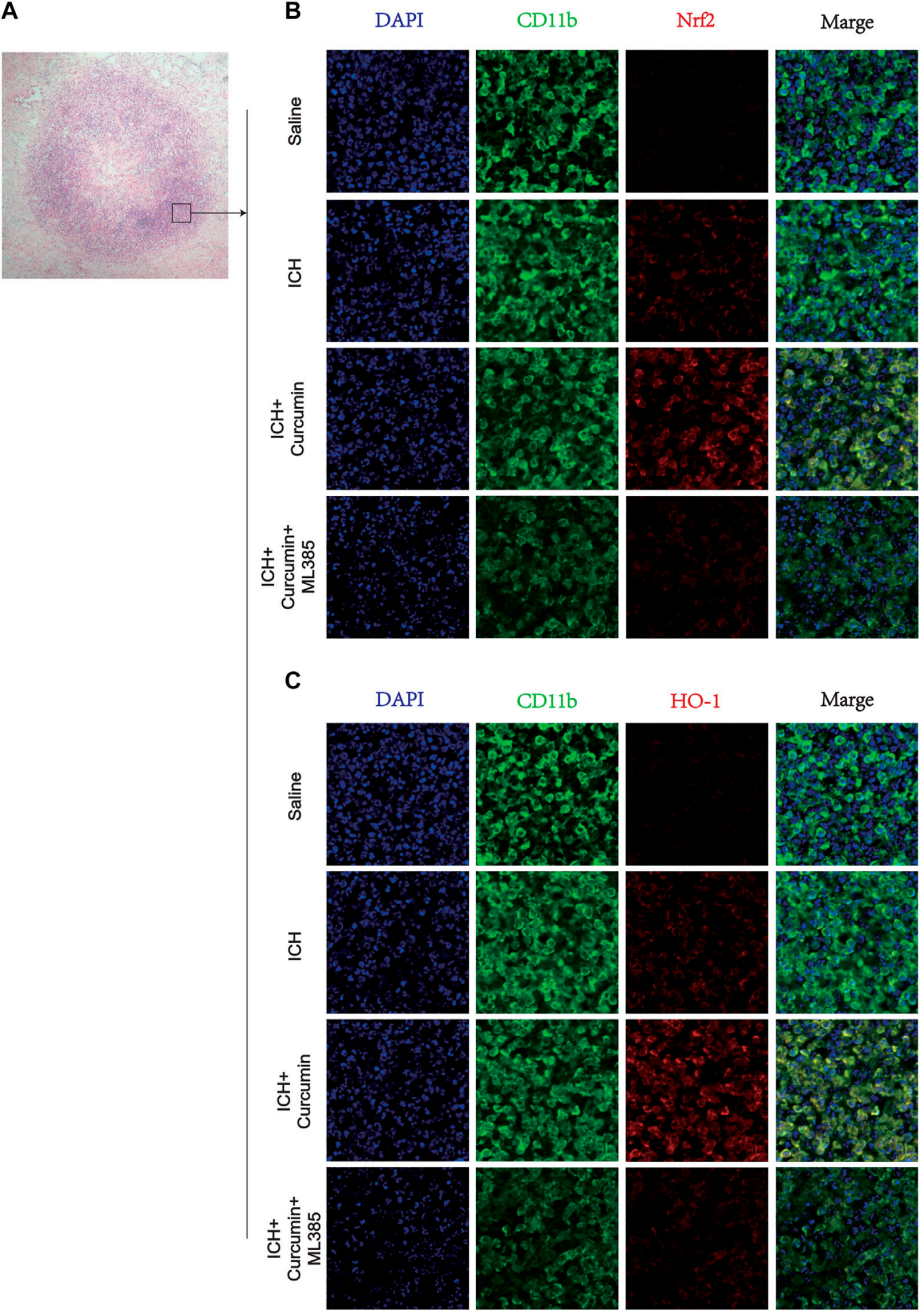
Immunofluorescence staining of microglia in the surrounding hematoma at 7 days post ICH. **(A)** H&E staining in the tissue surrounding the hematoma. **(B)** The microglial membrane protein CD11b was labeled with FITC, and the membrane protein Nrf2 was labeled with Texas Red. **(C)** The microglial membrane protein CD11b was labeled with FITC, and the membrane protein HO-1 was labeled with Texas Red. *n* = 3/group. The data are from at least three such independent experiments.

In further experiments, we explored the molecular mechanism of curcumin inhibiting intracranial oxidative stress response after ICH. At 7 days after ICH, the experiments detected ROS, MDA, and GSH levels in the brain tissue around the hematoma. And the results showed that curcumin could significantly reduce ROS and MDA levels in the brain tissue and significantly inhibited GSH depletion. However, when ML385 inhibited nrf2, curcumin lost its inhibitory effect on intracranial oxidative damage (Figure 7D). Furthermore experiments were conducted to explore the molecular pharmacological mechanism of curcumin. Western blot analysis of the brain tissue around the intracranial hematoma of rats 7 days after ICH showed that curcumin significantly increased intracranial Nrf2 and promoted the expression of downstream antioxidant factors (including HO-1, NQO1, Gpx4, and SOD). Inhibition of Nrf2 by ML385 significantly reduced curcumin activation (Figures 7A,B). Meanwhile, RT-qPCR was used to evaluate mRNA in brain tissues around hematoma to more accurately evaluate the promoting effect of Nrf2/HO-1 activated by curcumin on downstream gene expression. The results showed that curcumin promoted Nrf2 expression and increased the expression of downstream antioxidant genes, including HO-1, NQO1, Gpx4, and SOD (Figure 7C). The results suggest that curcumin can effectively activate the Nrf2/HO-1 pathway and promote the expression of a series of downstream antioxidant genes. Immunofluorescence staining of brain tissue around the intracranial hematoma in rats supported this conclusion (Figure 8B).

**FIGURE 9:**
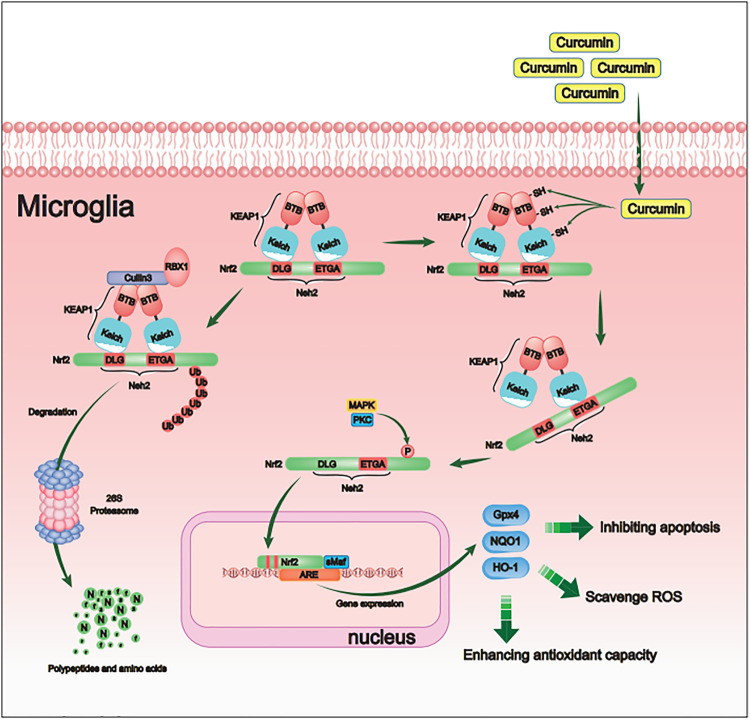
Schematic diagram of the molecular mechanism by which curcumin promotes antioxidant activity of intracranial microglia after intracerebral hemorrhage. By activating the Nrf2/ARE pathway, curcumin promoted the expression of many downstream antioxidant-related genes (including HO-1, NQO1, and Gpx4). Therefore, curcumin inhibited the cerebral oxidative injury after ICH.

## Discussion

Oxidative stress plays a crucial role in the pathological process of neurological injury after ICH (Hu et al., 2016; Wu et al., 2021). Secondary nerve damage after ICH is mainly due to mitochondria-dependent neuronal apoptosis caused by continuous oxidative stress (Duan et al., 2016), and various antioxidant cellular pathways may become new therapeutic targets to rescue neurons and promote functional nerve restoration during ICH treatment (Righy et al., 2016). In recent years, several studies have demonstrated that curcumin can promote the recovery of neurological function after ICH by inhibiting intracranial oxidative stress and regulating redox balance (Xie et al., 2020; Deng et al., 2021). Potent antioxidant drugs can effectively inhibit the rapidly increasing ROS during the early stage of ICH, thereby inhibiting neuronal necrosis (Zhang et al., 2016). However, long-term maintenance of the intracranial oxidative stress balance after ICH cannot rely solely on the direct scavenging of ROS. By activating a series of cellular pathways that regulate redox balance in intracranial glial cells, reactive oxygen species can be spontaneously cleared to maintain redox balance, which may contribute to the recovery of neurological function (Keep et al., 2012). Therefore, it is very effective in indirectly maintaining the redox balance in the central nervous system by regulating the Nrf2 pathway in glial cells (Zhao et al., 2007a). *In vivo* and *in vitro* studies have demonstrated that oral curcumin can inhibit the oxidative stress injury caused by ischemia—reperfusion, and this treatment can significantly reduce ROS in brain tissue and promote the expression of antioxidant genes (Subedi and Gaire, 2021). This outcome may be due to curcumin-mediated activation of the Nrf/ARE pathway, thereby inhibiting oxidative stress (Li et al., 2016). In this study, we used curcumin to treat a rat model of ICH in which autologous blood was injected into the striatum, and the results showed that curcumin significantly promoted the clearance of intracranial hematoma and alleviated perihematomal cerebral edema. The therapeutic effect of curcumin in animal models of ICH has been demonstrated in multiple studies over the past few years. In a 2015 study, it was demonstrated that curcumin alleviated perihematomal cerebral edema and reduced brain water content in a mouse model of ICH (Wang et al., 2015). Previously, curcumin was mainly used as an anti-inflammatory therapy and showed solid therapeutic potential in treating hepatitis and nephritis (Leclercq et al., 2004; Molina-Jijón et al., 2011). In another study, curcumin was shown to promote the phagocytosis of microglia, thereby promoting hematoma absorption and relieving perihematomal cerebral edema (King et al., 2011). Furthermore, curcumin inhibited neuroinflammation and the gene expression of IL-6, IL-1β, and TNF-α (Yang et al., 2014). This study suggests that ICH may have a potential therapeutic effect on ICH treatment. Therefore, it is important to clarify the relevant cellular pathways through which curcumin exerts its therapeutic effect.

*In vitro*, we used the HAPI cells and the primary cortical microglial cells of rats to confirm the agonistic effect of curcumin on Nrf2. After primary microglia were isolated, purified, and cultured for 2 to 3 generations, cell shape and proliferation rates were stabilized. Rat primary microglia and HAPI cells have similar morphology and proliferation rates and the same specific immune marker, Iba-1. The experimental results demonstrated that curcumin had similar activating effects on the Nrf2 pathway in the primary microglia and HAPI cells.

There are three main reasons for severe oxidative stress injury in the brain after ICH: 1) after the blood vessel ruptures, blood flows into the brain parenchyma to form a hematoma, which produces a mass effect in the brain; the hematoma causes ischemia and hypoxia in the brain tissue around the hematoma; the increase in ROS causes oxidative stress damage, resulting in cerebral edema around the hematoma (Keep et al., 2012); 2) oxygenated hemoglobin in the hematoma releases a large amount of ROS, causing oxidative stress injury to the brain tissue (Righy et al., 2016); 3) the protein with the highest content in the hematoma is hemoglobin, and heme is an important active substance; the heme molecule’s protoporphyrin ring of ferrous ions can participate in oxidation reactions and exchange electrons with various substrates, resulting in oxidative stress damage (Wan et al., 2019).

Previous studies have used heme-stimulated cells as an *in vitro* model to mimic intracranial oxidative stress injury after ICH (Duan et al., 2021). Two studies from 2018 to 2020 used heme to stimulate primary neuronal cells to mimic ICH-induced neuronal ferroptosis (Karuppagounder et al., 2018; Yang et al., 2020). Therefore, in the *in vitro* experiments, we used heme to stimulate microglia to simulate oxidative stress injury in rat brain tissue after ICH.

ML385, which is a specific inhibitor of the Nrf2 pathway, was used as an inhibitor in our study to verify curcumin-induced activation of the Nrf2 pathway. The Nrf2-specific inhibitor ML385 has been used as an inhibitor in many previous studies to study the Nrf2/ARE pathway. In 2016, a study validated the specificity of ML385 in inhibiting the Nrf2 pathway. To test whether ML385 interacts with NRF2 and affects the DNA-binding activity of the NRF2-MAFG protein complex, the researchers performed a fluorescence polarization assay using fluorescein-labeled ARE-DNA (Singh et al., 2016). The results showed that anisotropy decreased in a dose-dependent manner after adding ML385, indicating that the NRF2-MAFG protein complex had been isolated from fluorescein-labeled ARE-DNA (Singh et al., 2016). In our study, the results showed that when ML385 inhibited the Nrf2 pathway, the antioxidant therapeutic effect of curcumin was counteracted. The specific agonistic effect of curcumin on the Nrf2 pathway may be better explained if an Nrf2-knockout animal model is used. A 2018 study used Nrf2-knockout mice to create a traumatic brain injury (TBI) model and verified the agonistic effect of curcumin on Nrf2 (Dong et al., 2018). Another study in 2015 used Nrf2-knockout mice, and genetic sequencing was performed on the livers and small intestines of the mice after curcumin administration and it showed that curcumin activated Nrf2 and its downstream antioxidant genes (Shen et al., 2006). However, as an essential cellular pathway for maintaining redox balance *in vivo*, Nrf2 regulates the transcription of more than 200 downstream genes (Giudice and Montella, 2006). The Nrf2-/Nrf2-mice significantly impact experimental results due to the lack of key transcription factors that regulate redox balance. Therefore, in future studies, we will try to knock out the Nrf2 gene in microglia *in vitro* or specifically knock out the Nrf2 gene in rat intracranial microglia to verify that curcumin agonism is specific to Nrf2.

Keap1 is a key protein that regulates Nrf2 degradation *in vivo*. Under basal conditions, KEAP1 homodimers promote Nrf2 ubiquitylation, marking Nrf2 for proteasomal degradation (Wu and Papagiannakopoulos, 2020). Following NRF2 ubiquitylation, KEAP1 is recycled and binds to newly synthesized NRF2. Under oxidative conditions, key cysteine residues on KEAP1 are covalently modified, preventing it from mediating NRF2 ubiquitylation. Then, newly synthesized NRF2 can accumulate and translocate to the nucleus, where it dimerizes with one of the small MAF proteins to promote the transcription of cytoprotective genes (Kerins and Ooi, 2018). Curcumin has a specific activating effect on the Nrf2 pathway. After some chemical groups in curcumin react with the sulfhydryl group in Keap1, the hydrogen bond in the Keap1 protein is destroyed, thereby reducing the affinity of Keap1 for Nrf2 (Shehzad and Lee, 2013). Curcumin can accelerate meta-hydroxyl groups and the reactivity of benzylidene alkanones with sulfhydryl groups, and the associated increase in inducer potency for Nrf2 proteins may be explained by the enhanced reactivity of the sulfhydryl group through inductive hydrogen bonding of the neighboring phenolic hydroxyl group(s) (Dinkova-Kostova et al., 2001).

In conclusion, this study showed that Nrf2 is a critical cellular pathway by which curcumin reduces intracranial oxidative stress in the ICH rat model by inhibiting the affinity of Keap1 and Nrf2, inhibiting the degradation pathway of Nrf2, and promoting Nrf2 nuclear transfer. By activating the Nrf2/ARE pathway, curcumin promoted the expression of many downstream antioxidant-related genes (including HO-1, NQO1, and Gpx4). Curcumin inhibited the increase in ROS in microglia in the tissue surrounding the intracranial hematoma, alleviated lipid peroxidation, and inhibited glutathione depletion. Therefore, curcumin can promote the removal of intracranial hematoma, relieve cerebral edema around the hematoma and promote the recovery of neurological function after ICH.

## Data Availability

The original contributions presented in the study are included in the article/Supplementary Material, further inquiries can be directed to the corresponding authors.
