# Supplementary material for: Curcumin Restrains Oxidative Stress of After Intracerebral Hemorrhage in Rat by Activating the Nrf2/HO-1 Pathway
Source: Front Pharmacol. 2022 Apr 27;13:889226. doi: 10.3389/fphar.2022.889226 (PMC9092178; doi:10.3389/fphar.2022.889226)
Supplement: Supplementary file 3 [file Table1.DOCX]

The supplementary materials shared connection is as follows:

https://www.jianguoyun.com/p/Da1vdgsQm_juCRjozLAE

Supplementary materials contains：

1. Individual image files for your western blot gels.
2. The original files for any microscopy images.
3. Flow cytometry files (.fcs) included in your figures, and gating strategies for each flow experiment with clear labels to differentiate separate experiments.
