# Supplementary material for: Curcumin Restrains Oxidative Stress of After Intracerebral Hemorrhage in Rat by Activating the Nrf2/HO-1 Pathway
Source: Front Pharmacol. 2022 Apr 27;13:889226. doi: 10.3389/fphar.2022.889226 (PMC9092178; doi:10.3389/fphar.2022.889226)

Western Blot row pictures for Fig. 3A

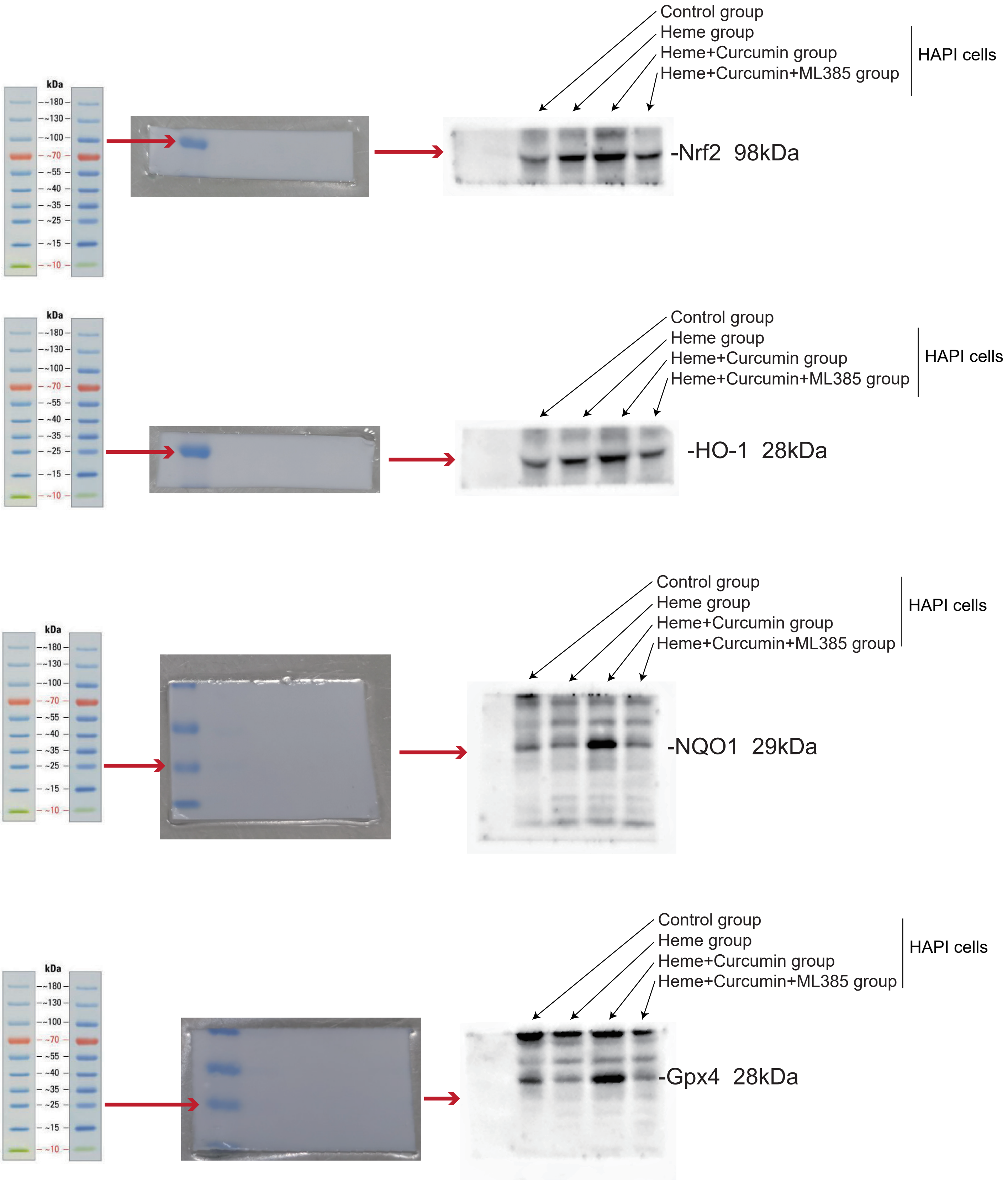

Western Blot row pictures for Fig. 3A (continued)

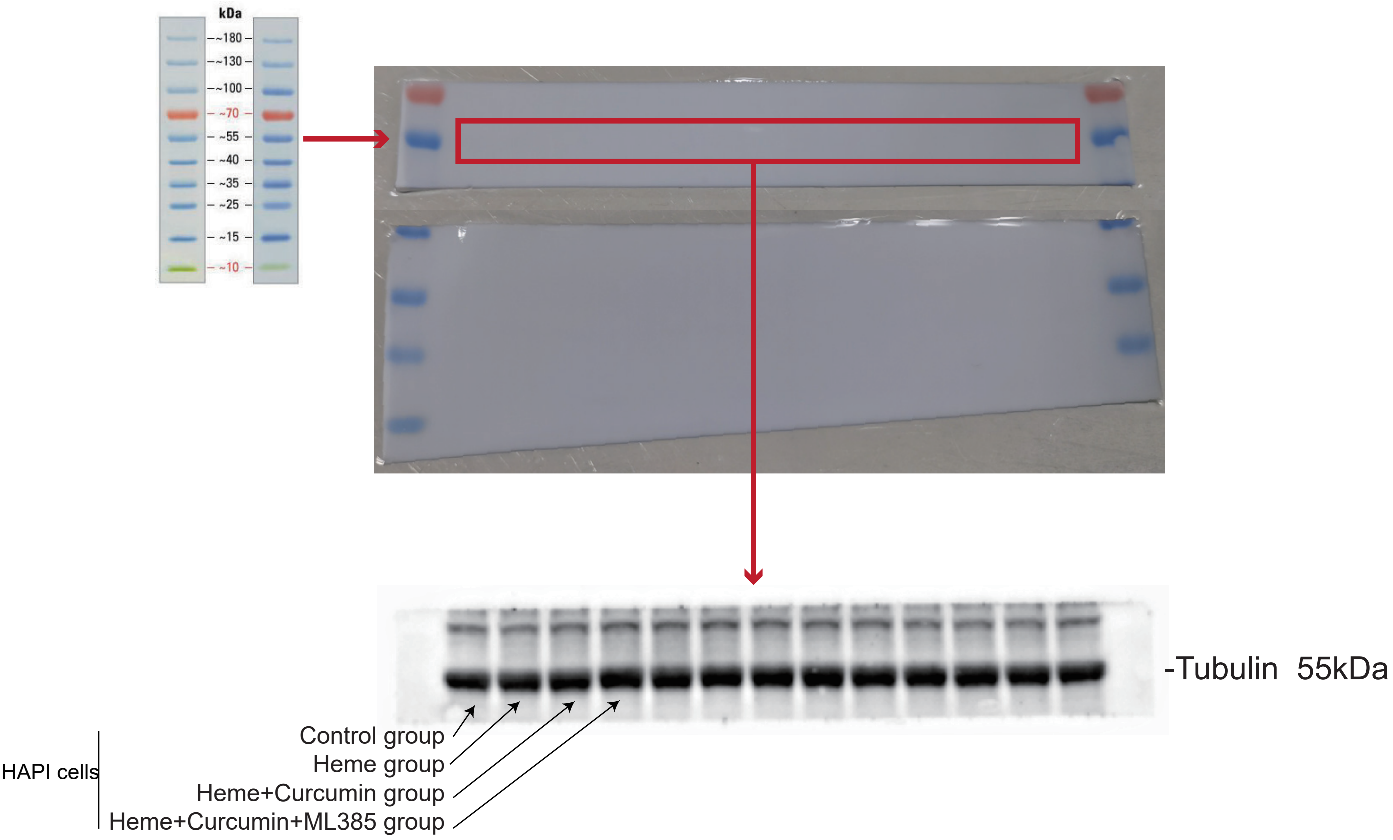

Western Blot row pictures for Fig. 5A

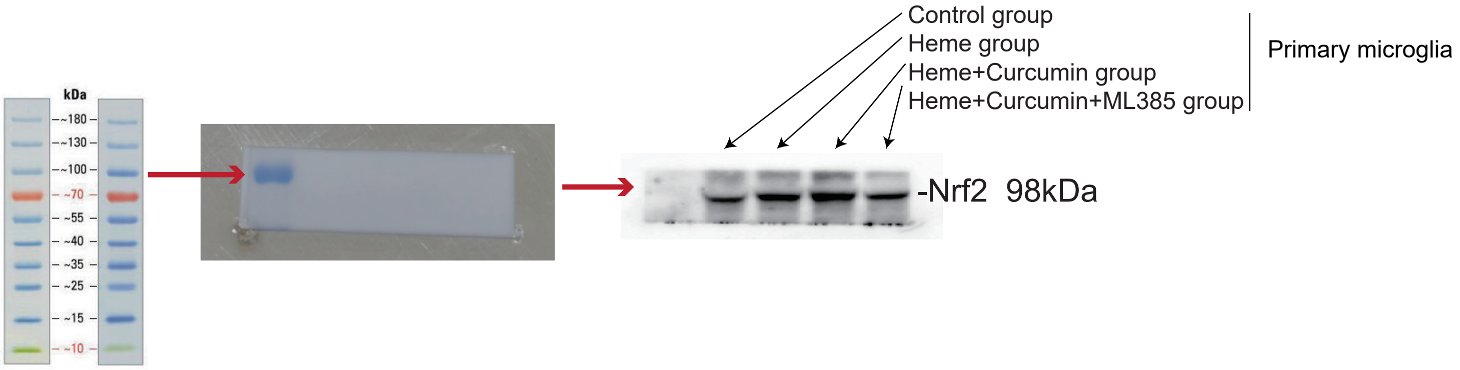

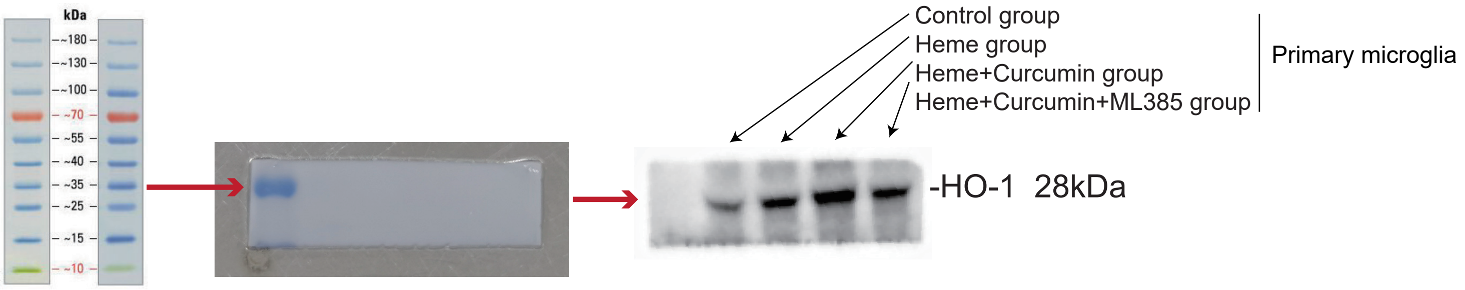

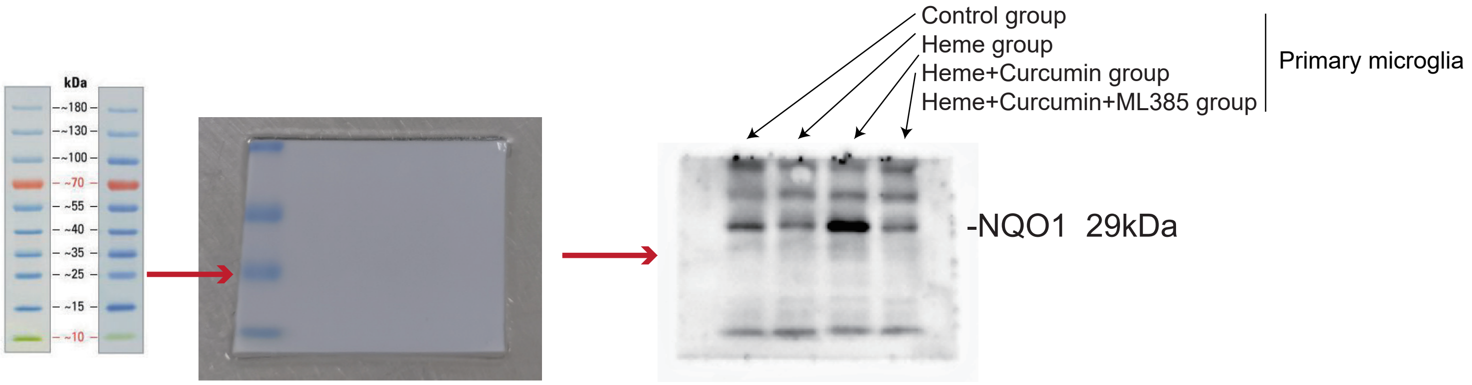

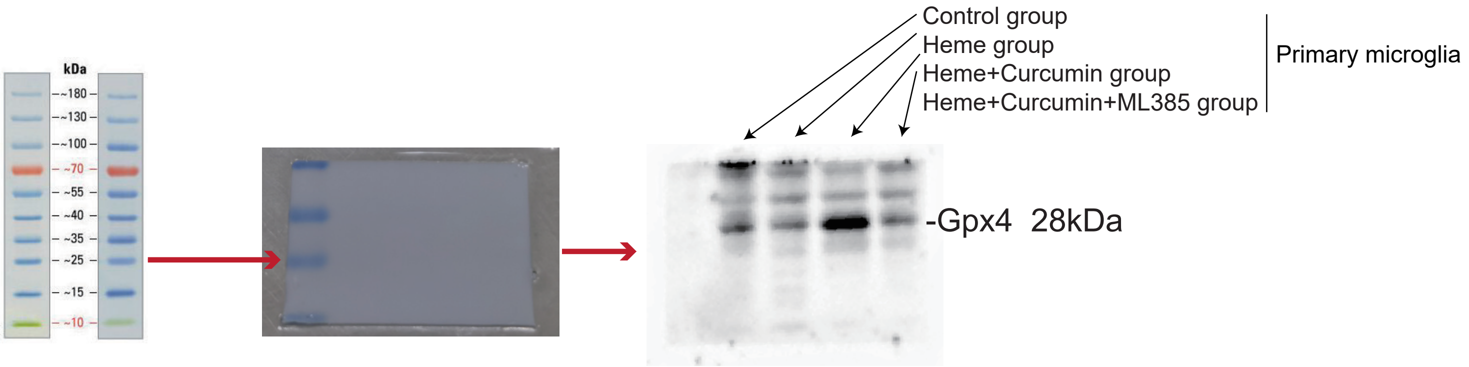

Western Blot row pictures for Fig. 5A (continued)

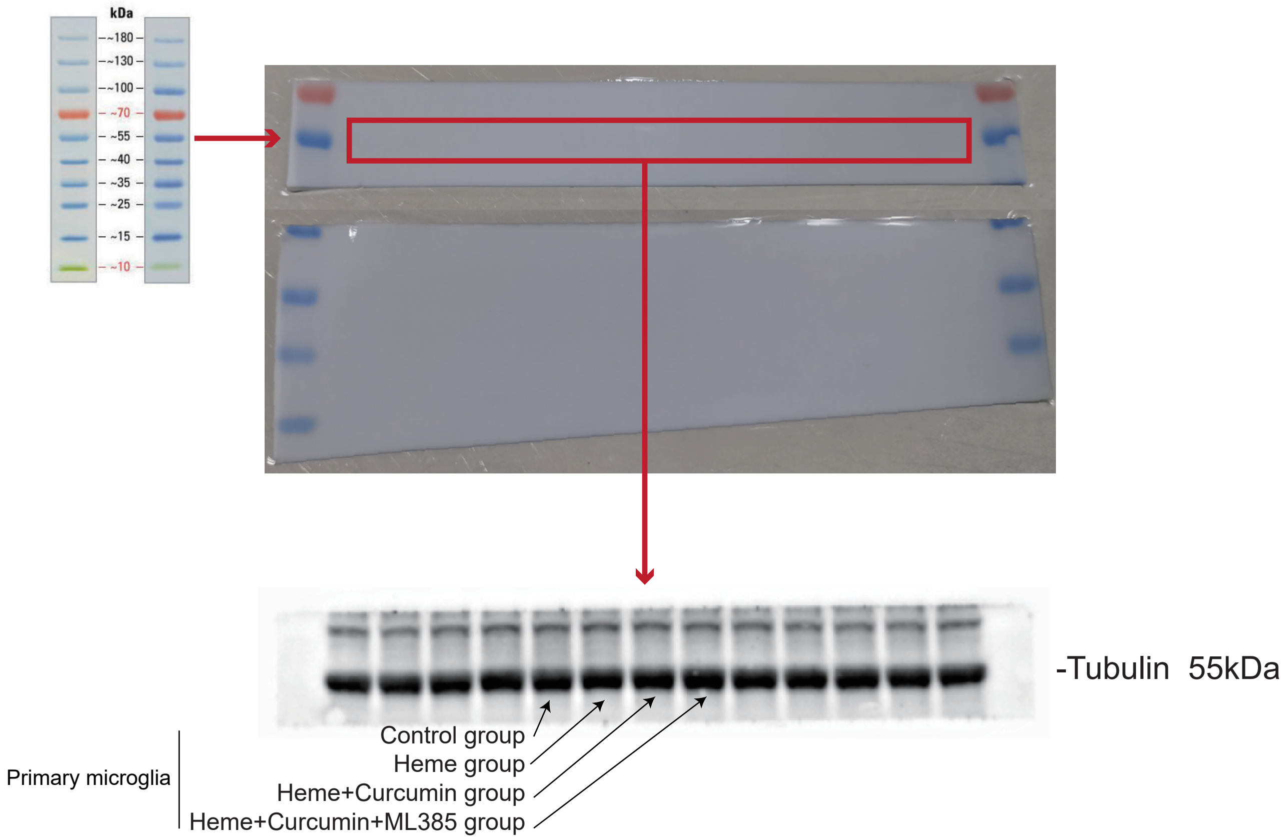

Western Blot row pictures for Fig. 8A

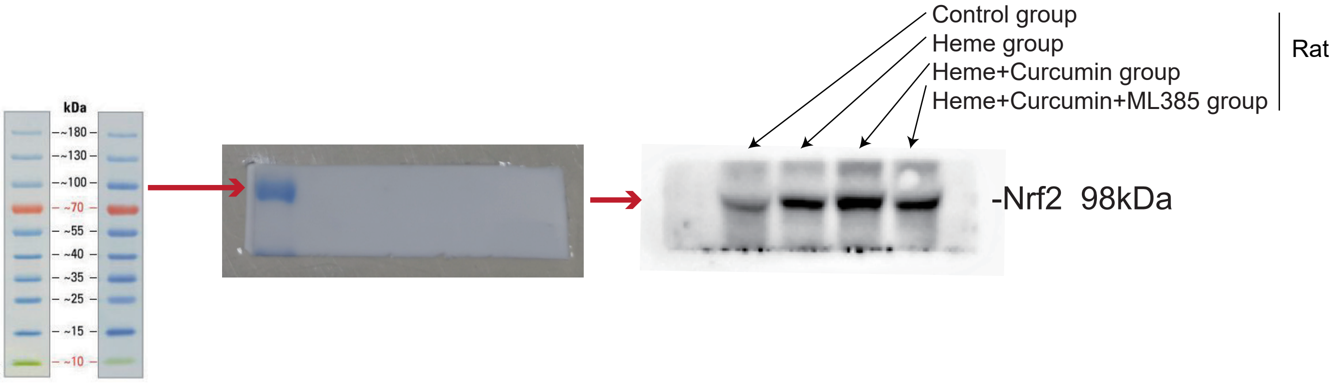

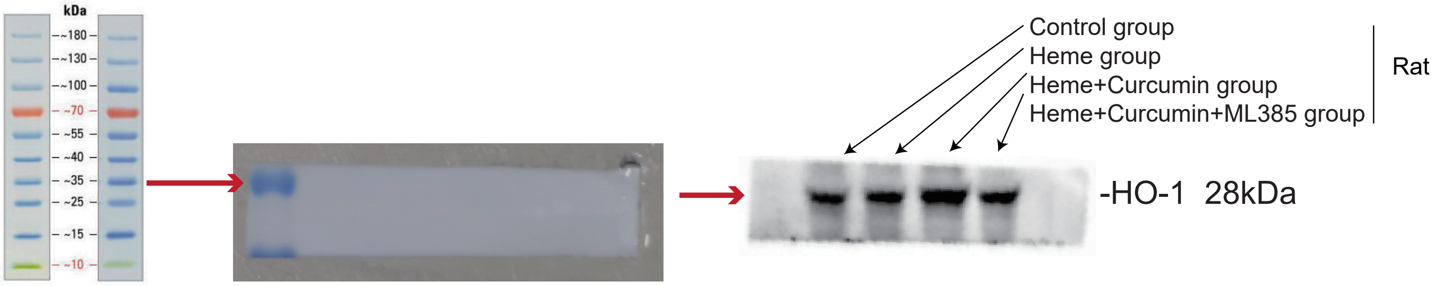

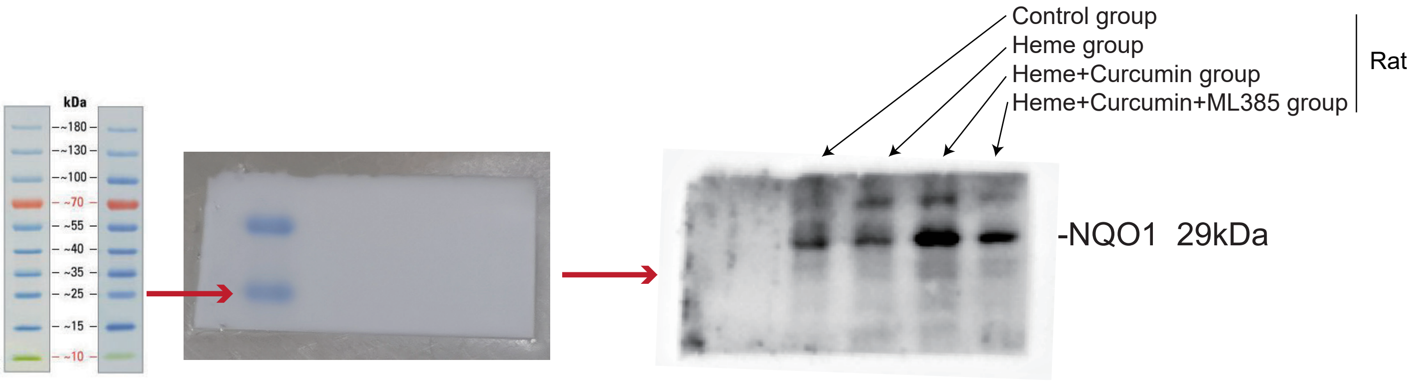

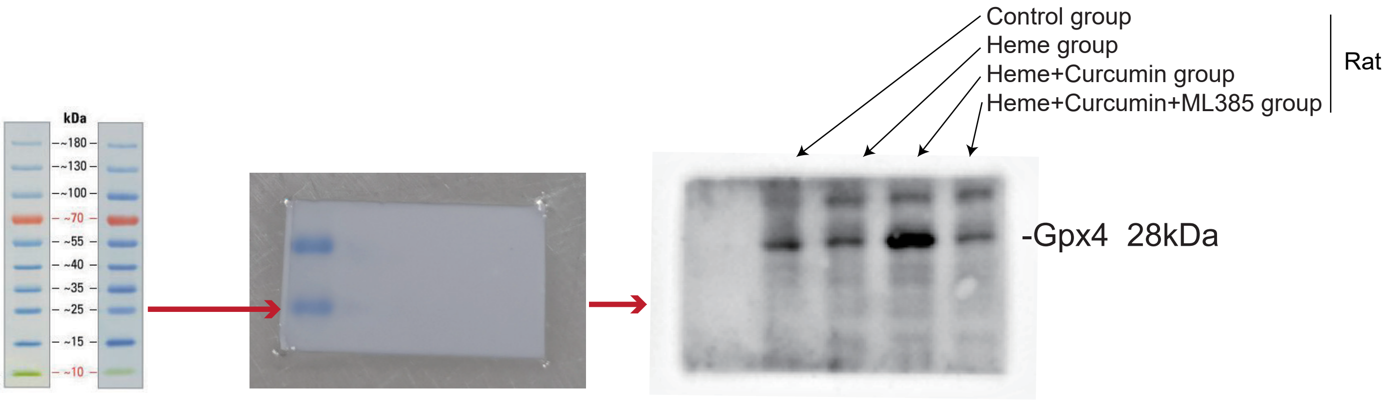

Western Blot row pictures for Fig. 8A (continued)

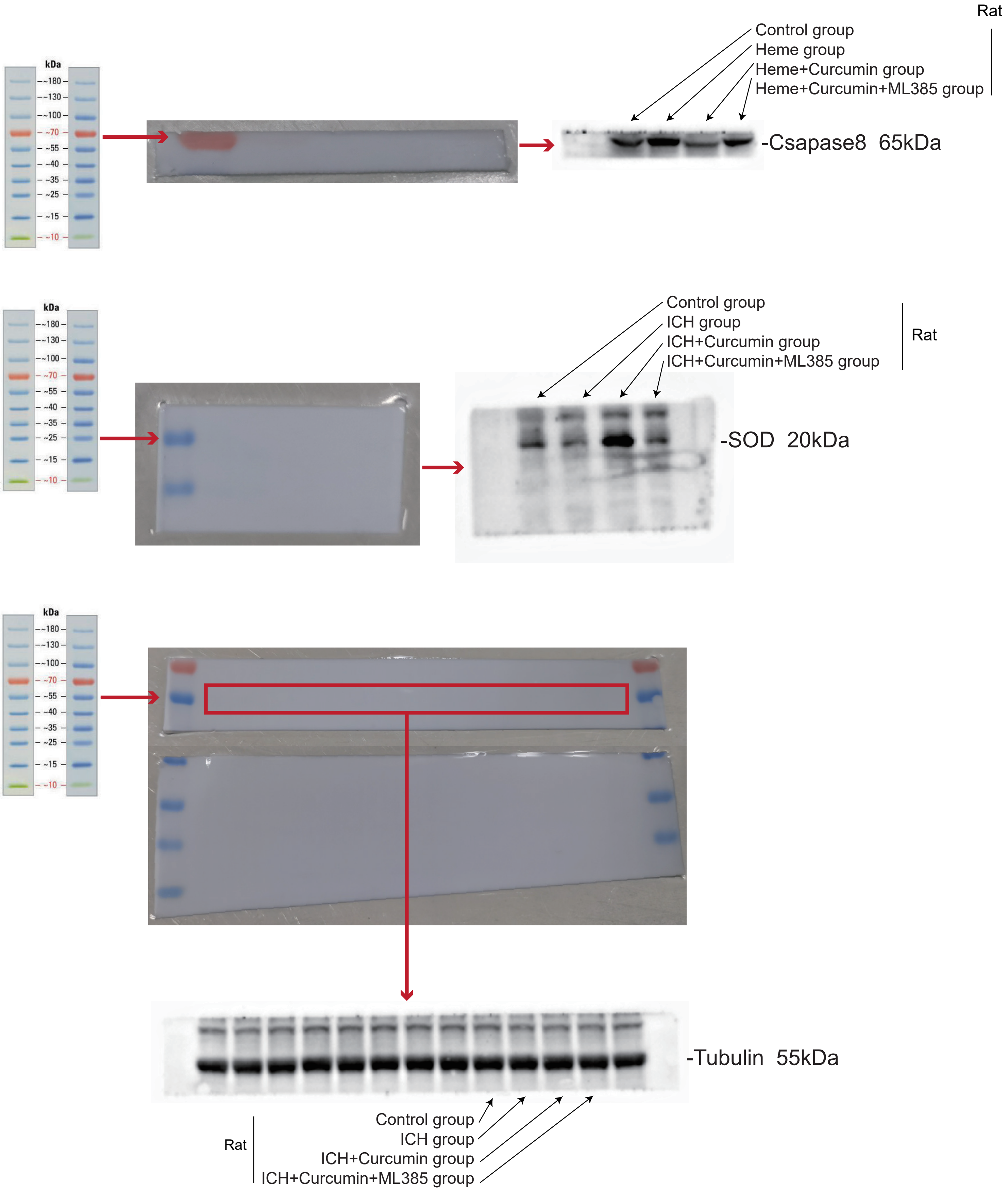

Supplement: Supplementary file 5 [file DataSheet1.PDF]
